# Cancer Drug Delivery Systems Using Bacterial Toxin Translocation Mechanisms

**DOI:** 10.3390/bioengineering10070813

**Published:** 2023-07-07

**Authors:** Linxiang Yin, Hatim Thaker

**Affiliations:** 1 Department of Urology, Boston Children’s Hospital, Boston, MA 02115, USA; 2 Department of Surgery, Harvard Medical School, Boston, MA 02115, USA; 3 Department of Microbiology, Harvard Medical School, Boston, MA 02115, USA

**Keywords:** bacterial toxin, translocation mechanism, drug delivery, cancer therapy, immunotoxins

## Abstract

Recent advances in targeted cancer therapy hold great promise for both research and clinical applications and push the boundaries in finding new treatments for various currently incurable cancers. However, these therapies require specific cell-targeting mechanisms for the efficient delivery of drug cargo across the cell membrane to reach intracellular targets and avoid diffusion to unwanted tissues. Traditional drug delivery systems suffer from a limited ability to travel across the barriers posed by cell membranes and, therefore, there is a need for high doses, which are associated with adverse reactions and safety concerns. Bacterial toxins have evolved naturally to specifically target cell subtypes via their receptor binding module, penetrating the cell membrane efficiently through the membrane translocation process and then successfully delivering the toxic cargo into the host cytosol. They have, thus, been harnessed for the delivery of various drugs. In this review, we focus on bacterial toxin translocation mechanisms and recent progress in the targeted delivery systems of cancer therapy drugs that have been inspired by the receptor binding and membrane translocation processes of the anthrax toxin protective antigen, diphtheria toxin, and *Pseudomonas* exotoxin A. We also discuss the challenges and limitations of these studies that should be addressed before bacterial toxin-based drug delivery systems can become a viable new generation of drug delivery approaches in clinical translation.

## 1. Introduction

Bacterial toxins are virulence factors that harm specific host cells by inhibiting cell growth and inducing cell death to favor bacterial infections that cause diseases in humans and animals [1,2,3]. Many bacterial toxins exert their toxic effects by targeting specific types of cells, entering the cells, and then interrupting key host intracellular cell signaling pathways [4]. The function of these bacterial toxins depends on their highly modular and efficient subdomains that can act as guided membrane translocation machinery; this includes the receptor binding domain, the translocation domain, as well as the catalytic domain. The receptor binding domain specifically targets host cell surface receptors and even host cell membranes, which enable the toxins to target various cell types, including neurons and immune cells [4,5,6]. The translocation domain confers the ability of toxins to become absorbed by the host cells. Additionally, the catalytic domain directly modulates host signaling pathways to inhibit host cell growth and even kill the host cells. The translocation domains of bacterial toxins, in particular, are an evolutionally powerful machine that can overcome the lipid bilayer barrier to deliver cargo into the host cells [7,8]. The overall translocation domains of bacterial toxins can be mainly divided into two classes, depending on the beta-sheet or the alpha-helix membrane integration elements. The former class of toxins is represented by the anthrax toxin, and the latter class is represented by the diphtheria toxin and botulinum neurotoxin [9,10,11,12]. The process by which bacterial toxins overcome the membrane barrier and achieve cargo delivery is an intricate and intriguing process involving a comprehensive and sequential series of events [4]. Understanding the precise molecular events during the membrane translocation of bacterial toxins is crucial for deciphering the cargo delivery process and reprogramming bacterial toxin translocation for various medical purposes, including targeted cancer drug delivery.

Cancer is one of the leading causes of human death worldwide each year and is characterized by abnormal growth and uncontrollable expansion of cells. Despite great improvements in the treatment of cancer, it is still one of the top diseases that threaten human health [13]. It is still difficult to target and treat certain types of cancers because it is especially challenging to target and deliver the drugs to certain cancer cell types [14]. Bacterial toxins are a naturally evolved protein machinery that can target and deliver a toxic cargo to disrupt specific types of cells. Thus, harnessing the cell-specific transmembrane delivery properties of bacterial toxins to treat cancer is a promising strategy for the intracellular delivery of various drugs. Bacterial and plant toxins attached with cell-specific targeting monoclonal antibodies have been developed to kill cancer cells. These antibody-toxin bi-functional molecules are called immunotoxins (ITs), which are composed of antibodies that are produced by immune systems linked to toxins [15,16]. Several bacterial toxin-based immunotoxin cancer drugs have been approved, and more immunotoxin prodrugs are now under clinical trials [17,18,19]. In this review, we mainly focus on recent progress in the membrane translocation mechanisms of the anthrax toxin protective antigen (PA), diphtheria toxin, and *Pseudomonas* exotoxin A and the related cancer-targeting immunotoxins that are inspired by their receptor binding and membrane translocation processes.

## 2. Anthrax Toxin PA-Based Cancer Drug Delivery

### 2.1. Anthrax Toxin

**Figure 1:**
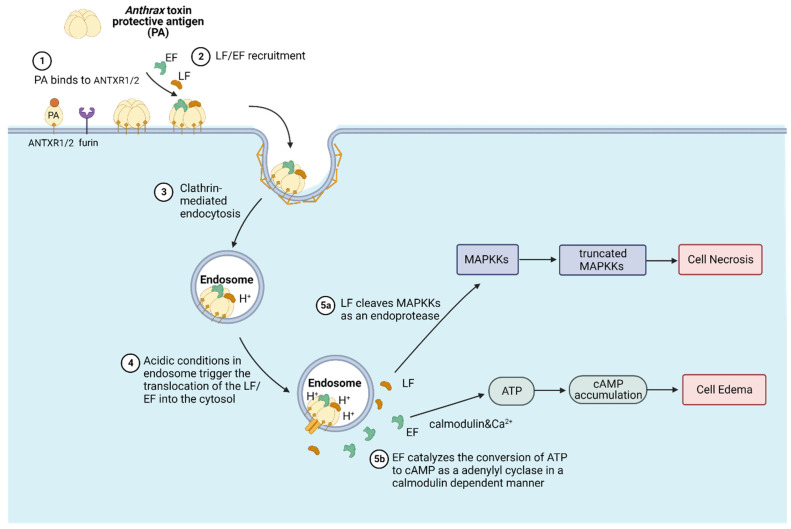
Anthrax toxin translocation mechanism. The anthrax toxin protective antigen (PA) first binds to the host cell membrane protein receptor anthrax toxin receptor ANTXR1/2 and then is cleaved by the host cell surface furin family protease to oligomerize. Then, the lethal factor (LF) and edema factor (EF) are recruited by PA oligomers, and the toxin complex is internalized by the host receptor-mediated endocytosis pathway. Acidic conditions within the endosome trigger the structural rearrangement and channel formation of PA for LF/EF translocation into the cytosol. Refolded LF cleaves cytosol target protein MAPKKs from the N-terminal, and refolded EF catalyzes the cAMP formation, thus inducing cell necrosis and edema, respectively. This image was adapted from “Mechanism of Action-Diphtheria Toxin” by BioRender.com (2023), accessed on 9 May 2023. Retrieved from https://app.biorender.com/biorender-templates, accessed on 9 May 2023.

Anthrax toxin is the major virulence factor for *Bacillus anthracis* and is the causative agent of the severe disease called anthrax. It is a binary toxin that consists of the receptor-binding and translocation machinery protective antigen (PA) plus enzymatic executor factors, which are referred to as the lethal factor (LF) and edema factor (EF) (Figure 1) [20]. The mechanism by which anthrax toxins exert their toxic effect on the host cell involves a series of sequential steps, which are summarized as follows: 1. Anthrax toxin PA specifically targets host cell membrane proteins called anthrax toxin receptor 1 (ANTXR1) as well as anthrax toxin receptor 2 (ANTXR2) [21,22,23]. Then, the 83 kDa PA monomer (PA83) is cleaved by the cell surface furin family protease to form an active form 63 kDa PA monomer (PA63). 2. Furin protease cleavage and PA63 oligomerization provide interfaces for LF and EF binding and create the pre-channel for LF and EF’s further translocation. The PA63 heptamer is a more prevalent oligomerization state than the HA63 octamer on the host cell surface, even though the PA63 heptamer is less stable and more prone to form a premature channel than the PA63 octamer under physiologic temperatures and pH conditions. One explanation is that the host extracellular PA receptor drives the PA oligomerization and stabilizes the PA63 pentamer [24,25]. 3. The anthrax toxin complexes then become endocytosed by the host clathrin-mediated pathway. 4. Endosome acidification triggers the membrane insertion as well as the anthrax PA channel formation, which mediates the transmembrane delivery of LF and EF. 5. After endosome translocation, refolded anthrax toxin LF becomes a protein endoprotease that cleaves the N-terminal fragment of mitogen-activated protein kinase kinases (MAPKKs) and deactivates these kinases, leading to altered downstream signaling and cell apoptosis. Anthrax toxin EF is a calmodulin and Ca^2+^-dependent adenylyl cyclase. Refolded EF catalyzes the conversion of ATP to cAMP and induces the accumulation of intracellular cAMP, which can lead to impaired water homeostasis and edema [26].

### 2.2. Anthrax Translocation Mechanisms

**Figure 2:**
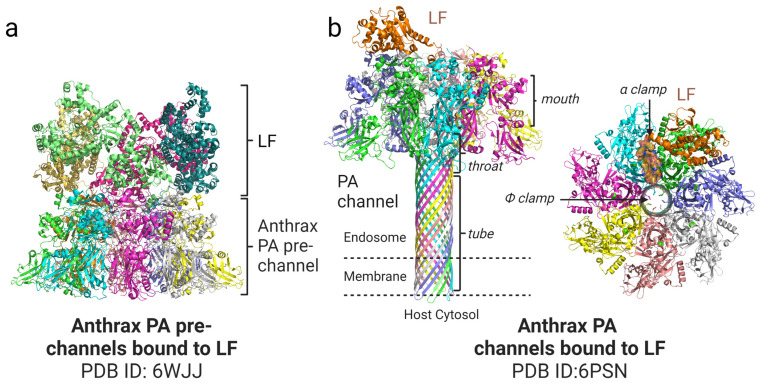
Cryo-EM structure models of Anthrax PA prechannel-LF and PA channel–LF complexes. (**a**) Ribbon representation of Anthrax PA_8_ prechannel–LF_4_ complex viewed from the side and colored by subunits. (**b**) Ribbon representation of Anthrax PA_7_ channel–LF complex viewed from the side (**left**) and top (**right**) and colored by subunits. All the protein structural models were generated using the program PyMOL (https://pymol.org/2/ (accessed on 13 March 2023).).

As the membrane translocation module of the anthrax toxin, PA mediates the delivery of LF and EF through the membrane barrier into the host cytosol. LF and EF bind to the oligomeric HA63 pre-channel, forming the “flowers-in-vase” conformation, where the flowers correspond to the LF or EF cargo and the vase corresponds to the oligomeric HA (Figure 2a) [27]. The anthrax toxin complex hijacks the endocytosis process and enters the endosome, which then becomes acidified. The PA pre-channel is then triggered by the endosome’s low pH to form a membrane-inserted pore structure that contains an ion-conductive channel for the cargo transport (Figure 2b) [9].

According to the cryo-EM structure of the PA channel, the pore architecture of PA is a mushroom-like object with a 7.5 nm long and 12.5 nm diameter cap and a stem that is 10.5 nm long and 2.7 nm in diameter. During channel formation, the PA domain 2 disordered 2β2-2β3 loops together with the flanking loops generating a long β barrel that inserts into the membrane and forms a channel that is embedded in the lipid bilayer. This transmembrane channel only allows the translocation of unfolded LF or EF [9,28]. MOLE toolkit analysis for the characterization of channel macromolecular structures shows that the PA channel could be divided into four parts from the top to the bottom: α clamp containing mouth, Φ clamp, negatively charged throat, and the tube (Figure 2b). The translocation of the cargo starts from the channel mouth near the α clamp, a hydrophobic groove created by two nearby protomers to nonspecifically bind to the cargo protein α helix. The narrowest part of the channel is the Φ clamp, with a diameter of 6 angstroms formed by the Phenylalanine 427 (Phe427) residues contributed by each PA protomer, which is just large enough to pass through the unfolded α-helix but not large enough to accommodate the well-folded protein [9]. Since the cargo should be unfolded to proceed through the Φ clamp, highly stable cargo is not able to be translocated efficiently [4,29]. The ^19^F NMR study, with the site-specific labeling of the Phe427 residues with p-fluorophenylalanine (pF-Phe427), shows that pF-Phe427 is intrinsically dynamic in the pre-channel state and even more dynamic in the channel state. Such dynamic behavior of the Φ clamp could provide flexibility and room for unfolded polypeptide chain movement during cargo translocation [30]. The mouth on the top and the tube on the bottom are the opening of the channel, while the Φ clamp seals the channel to ensure that it is impermeable to small molecules before and during the cargo translocation. In contrast to the largely hydrophilic inner surface of the channel, the outer surface of the PA channel is largely hydrophobic, which could contribute to the binding of the hydrophobic lipid bilayer and stabilize the transmembrane channel [9].

PA63-bound LF or EF unfolding is induced by endosome acidification [31]. The N-terminus of PA63-bound LF or EF then enter the PA channel and initiate the entry of cargo into the PA channel [20]. In the presence of a pH gradient or membrane potential, the PA channel serves as an active transporter and moves the cargo to further the N-to-C translocation [28,31]. A charged state-dependent Brownian-ratchet mechanism, with the help of molecular chaperones in concert with the translocation process, leads to successful and efficient transmembrane cargo delivery [32,33]. Acidic amino acid residues within a PA cavity are mostly protonated and positively charged. Once exposed to the cytosol, the cargo residues are more negatively charged. Since the inner cavity of the channel is negatively charged, the negatively charged residues translocated out of the channel could not move back due to the electrostatic repulsion force, thus ensuring the unidirectional movement of the translocating cargo [9,29].

### 2.3. Anthrax Toxin PA-Based Drug Delivery for Cancer Therapy

The anthrax toxin tripartite system is versatile for the drug delivery of enzymatic moieties into cells. In 1992, Naveen Arora and Stephen H. Leppla et al. first reported that *Pseudomonas* exotoxin A ADP-ribosylation domain and the LF fusion protein could be delivered into the cytosol of mammalian cells by anthrax PA. This discovery opened a new frontier with regard to the use of anthrax toxin PA as a drug delivery system for various non-native cargoes [34]. Later discoveries have showed that the N-terminal sequences of PA initiate the translocation, and the N-terminal sequences of LF (LFn) are required to deliver the peptide into the cytosol [20,35]. Thus, various cancer cell-killing cargoes can fuse with LFn, and then be guided by LFn to translocate through the PA channel into the cytosol (Table 1).

Native PA mostly targets cells that express ANTXR1 and ANTXR2. To alter the targeting of these cells, PA domain 4 can be mutated (mPA) to ablate the binding to native receptors and then become fused with EGF (mPA-EGF) to target cancer cells that express the EGF receptor [36]. The conjugated mPA-EGF triggered apoptosis in EGFR-expressing bladder cancer cells within about three minutes of toxin exposure time. Additionally, upon mPA-EGF treatment, decreases in the tumor mass were consistently observed in six tested dogs with a treatment-resistant bladder. In tumor-free mice and dogs, mPA-EGF induced no toxicity [37]. Additionally, PA could be engineered to fuse with a HER2 high-affinity affibody (mPA-ZHER2) to deliver various cytocidal effectors into trastuzumab-resistant HER2-positive tumor cells and induce cell death [38].

To further decrease this toxin’s off-target effects, the high-specificity tumor-targeting of anthrax-based drug delivery is required. Numerous proteases that enable tumor invasion and metastases are highly expressed in cancer cells and can be utilized for the cell-specific activation of anticancer pro-drugs. The furin cleavage site of PA could, thus, be mutated to sequences cleaved by proteases (such as matrix metalloproteinase and urokinase) that are highly expressed in target tumor cells [39,40,41]. Such an approach could synergize with cell-specific targeting moieties to further reduce non-specific toxicity to healthy cells and decrease the off-target adverse effects [42].

**Table 1:** Anthrax toxin PA-based cancer prodrugs.

Toxin/Toxin Fragment	Targeting Moiety	Target Cancer Cells or Diseases	Obtained Outcome	References
C-terminus of PA	c-Myc	c-Myc-specific hybridoma cell line	Mouse macrophages and c-Myc-specific hybridoma cell killing	[43]
Mutant PA (PA N682A D683A)	EGF	EGFR positive Human A431 epidermoid carcinoma cells	Enzymatic effector proteins transported into A431 carcinoma cells	[36]
Mutant PA (mPA)	HER2 Affibody	HER2 positive breast cancer cell lines	Specific killing of HER2 positive breast cancer cell lines; no off-target killing of HER2-negative cells	[38]
Zymogen activation PA	ANTXR1/2	Ovarian tumor cell lines	Selective killing of ovarian tumor cells; inhibition of ovarian tumor growth in preclinical xenograft models	[42]

## 3. Diphtheria Toxin Translocation Domain-Based Cancer Drug Delivery

### 3.1. Diphtheria Toxin and Its Mechanism of Translocation

**Figure 3:**
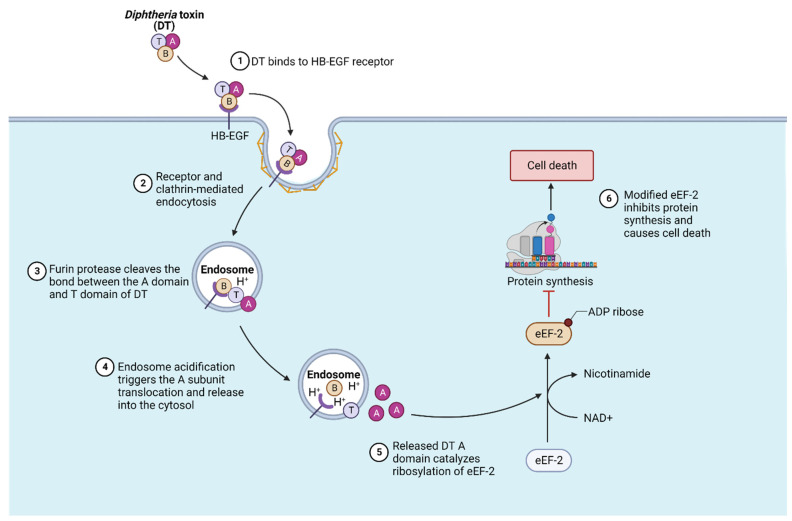
Diphtheria toxin translocation mechanisms. Diphtheria toxin (DT) binds to the cell surface receptor HB-EGF receptor and then becomes internalized via clathrin-mediated endocytosis. Within the endosome, the proteases partially cleave the bond between the DT domains. Endosome acidification triggers the translocation of the A subunit of DT into the cytosol. A subunit of DT catalyzes the ribosylation of the eukaryotic EF-2 (eEF-2) protein, which inhibits the host cell protein synthesis and thus induces cell death. This image was adapted from “Mechanism of Action-Diphtheria Toxin” by BioRender.com (2023), accessed on 1 May 2023. Retrieved from https://app.biorender.com/biorender-templates, accessed on 1 May 2023.

The Diphtheria toxin (DT) is a highly potent single-chain diphtheria-causing toxin that is produced by Corynebacterium diphtheriae with a lysogenic beta phage [44,45]. It is a short AB-type toxin that consists of a catalytic A subunit plus the receptor-binding and membrane translocation B subunit. The crystal structure of the Diphtheria toxin reveals a Y-shaped architecture with a cytotoxic enzymatic domain (A domain), a receptor-binding domain (B domain) on top, and the translocation domain (T domain) on the bottom [11]. The B domain first binds to the host cell receptor heparin-binding EGF-like growth factor (HB-EGF) and then becomes endocytosed by the host endocytosis pathway into an endosome. Then, the endosome’s low pH facilitates the structural rearrangement of the T domain as well as the membrane translocation of the A domain into the cytosol (Figure 3). Once there, the A domain refolds and targets eEF-2 through the addition of ADP-ribose, which subsequently inhibits protein synthesis and leads to cell death [46,47,48].

The T domain of DT is mainly composed of a helical architecture [11]. The acidic environment within the endosome induces the partial unfolding of the T domain and the formation of a molten globule. During translocation, the T domain is triggered by the endosome acidic pH, and a loop in between helix 8 and helix 9 initiates the endosome membrane interaction and insertion of the T domain upon the protonation of the residues glutamic acid 349 (Glu349) and aspartic acid 352 (Asp352). In addition, the proline 345 (Pro345) at the end of helix 8 is also critical for mediating the membrane insertion of the T domain [49,50,51,52]. At least two hydrophobic helical segments are then inserted into the endosome membrane to form the channel for A domain translocation. This is referred to as the “double dagger” model for DT translocation. The helical “double dagger” motifs (the T domain hydrophobic helices 5–9) are very conserved [11,51,53].

### 3.2. Diphtheria Toxin T Domain-Based Drug Delivery

Compared with other bacterial toxins, diphtheria toxin is a readily expressed and extremely potent toxin that has minimal adverse effects on humans; it is thus widely used to selectively treat various cancers. Replacing the B domain with various cancer antigen-targeting antibodies or growth factors can successfully achieve tumor cell-specific targeting and tumor cell killing (Table 2). For example, interleukin-2 (IL-2) is an important immunomodulatory cytokine, mainly produced by CD4-positive (CD4+) T cells, and thus can be utilized to target some tumor cells that overexpress interleukin-2 receptor (IL-2R). A diphtheria toxin in which the B domain is truncated (DAB486) was fused with IL-2 to form a recombinant protein called DAB486IL-2 [54,55]. A subsequent shorter version of the recombinant protein DAB389IL-2 showed reduced immunogenicity and an increased half-life of the recombinant protein [56]. In a cell toxicity assay, DAB389IL-2 showed at least 100 times lower half maximal inhibitory concentrations (IC50s) to hematopoietic tumor cells expressing high affinity IL-2R than cells expressing low-affinity IL-2R. Success in clinical trials for the treatment of persistent and recurrent cutaneous T-cell lymphoma (CTCL) led to the FDA approval of DAB389IL-2 (denileukin diftitox or ONTAK^TM^) in 2008. However, ONTAK^TM^ suffered from production issues due to its *E. coli* expression system. It also had a severe side effect of vascular leak syndrome and was thus discontinued in 2014. The following studies show that ONTAK^TM^ from diphtheria toxin-resistant yeast or *C.diphtheria* expression systems have higher activity and purity than that from *E.coli* [46,57]. In addition, vascular leaks can be reduced by mutated versions of immunotoxins [58,59]. Similarly, since IL3-R is highly expressed in blastic plasmacytoid dendritic cell neoplasm (BPDCN) cells, DT388IL-3 was developed to selectively kill IL3-R overexpressing dendritic cell neoplasm cells [17,60]. The clinical trial results on patients with BPDCN have shown major responses, including complete response (CR) and partial response (PR), which has led to the FDA approval of DAB388IL-3 under the commercial name of Tagraxofusp^TM^ in 2018 [19].

## 4. *Pseudomonas* Exotoxin A Translocation Domain-Based Cancer Drug Delivery

### 4.1. Pseudomonas Exotoxin A and Its Translocation Mechanism

*Pseudomonas* exotoxin A (PE) is a highly potent toxin that is secreted by *Pseudomonas aeruginosa*. It is a single-chain multidomain AB toxin made up of an enzymatic A fragment and a cell-binding B fragment. The B fragment of PE specifically binds to the host cell receptor LRP1 (low-density lipoprotein receptor-related protein 1, or α2-macroglobulin), and then this toxin is subsequently internalized by clathrin-coated vesicles-mediated endocytosis. After furin cleavage and protein disulfide isomerase reduction, the cleaved PE fragment (in the late endosome) reaches the trans-Golgi network via the Rab9-regulated pathway and then the ER by KDEL-receptor pathway in a retrograde manner [67,68,69,70,71,72]. Alternatively, receptor-bound PE, with the help of the detergent-resistant membrane microdomain (lipid rafts) and caveolae-mediated endocytosis, hijacks the lipid-dependent sorting pathway to reach ER directly. Then PE utilizes the conserved cellular quality control ER-associated protein degradation pathway to move into the cytosol [73]. Once translocated, the catalytic A fragment subsequently inhibits the function of eukaryotic elongation factor-2 (eEF-2), which is critical for host protein synthesis through its ADP-ribosyltransferase activity using NAD+. This mechanism is very similar to that used by the Diphtheria toxin [74].

### 4.2. Pseudomonas Exotoxin-Based Cancer Drug Delivery

As one of the most potent bacterial toxins, PE-based immunotoxins for cancer treatment have also attracted intensive investigation and gained remarkable success. To minimize the protein size and reduce immune clearance, PE40 and PE38 have been created by removing the native receptor binding domain of PE. Then the truncated versions of the PE were linked to various targeting moieties such as antibodies, antibody fragments, or ligands (Table 3) [75]. As a successful example, Moxetumomab pasudotox (FDA approval: 2018) is a recombinant protein of PE38 that is fused with the disulfide stabilized variable fragment (dsFv) of the monoclonal antibody RFB4 against CD22. Since CD22 is an inhibitory BCR (B-cell receptor) co-receptor that is highly expressed in malignant B cells such as hairy cell leukemia (HCL), Moxetumomab pasudotox showed high specificity as well as high toxicity toward HCL tumor cells [76]. In addition, with an improved version of the original RFB4 antibody with higher CD22 affinity and the improvement of the Moxetumomab production process, Moxetumomab pasudotox showed remarkably enhanced IT activity, higher HCL efficacy and reduced toxicity in clinical trials [77]. In late 2018, Moxetumomab pasudotox (Lumoxiti^TM^) was approved by the US FDA as a treatment for adult patients with HCL refractory to prior systemic chemotherapy [18]. Moxetumomab pasudotox was approved by the European Medicines Agency (EMA) for HCL treatment in December 2020. However, Moxetumomab pasudotox still shows adverse effects such as capillary leakage syndrome and decreased renal function. Such side effects are mostly due to the non-specific targeting of Moxetumomab pasudotox to normal cells. Efforts have been made to generate less immunogenetic versions of IT mutants with less binding to normal cells [58,78]. Besides Moxetumomab pasudotox, PE has been fused with interleukin 13 or antibodies targeting CD326 (EpCAM), EGFR, and mesothelin for the treatment of various types of tumors. These ITs are still in clinical trials or have been discontinued due to either severe side effects or low efficacy. Given the FDA and EMA-approved Moxetumomab pasudotox for HCL treatment and continuous efforts to reduce the immunogenicity and off-targeting of PE-ITs, PE-based ITs are still a promising field for targeted cancer therapies [79,80,81].

## 5. Discussion

Intracellular proteins and signaling pathways represent vast drug targets, yet the cell membrane is a formidable barrier that prevents drugs from reaching their intracellular targets. Various drug delivery approaches are now being developed and optimized to overcome this challenge, including adeno-associated virus vectors, lipid nanoparticles, toxin proteins, endosymbiotic bacterial extracellular contractile injection systems (eCISs), and homologs of capsid protein-based platforms [85,86,87]. Among these, bacterial toxins have evolved by nature to efficiently penetrate the cell membrane and successfully deliver effector proteins into the host cytosol. Compared with systemic delivery systems, bacterial toxin-based targeted delivery systems are poised to minimize the off-target accumulation of drugs and thus have lower side effects. In recent decades, various bacterial toxin-based anti-cancer drugs have been designed and developed for targeted cancer therapy. Numerous tumor cell targeting moieties have also been optimized to increase targeting specificity and avoid general systemic diffusion. Among them, tamed Anthrax PA, Diphtheria toxin, and PE-based immunotoxins have been demonstrated to specifically deliver toxic cargoes into cells efficiently and cure previously hard-to-treat cancers. Nonetheless, the off-target effects remain a concern in current bacterial toxin-based therapies. A common off-target side effect is the capillary leak syndrome. When a toxin is administered intravenously, it enters the tissue from the capillary bed and can nonspecifically kill capillary endothelial cells. Thus, plasma fluid often leaks from the damaged capillary bed into nearby viscera, causing hypotension and fluid retention. Such off-target toxicity can be managed conservatively with hydration and steroids in the hope that the capillary leak syndrome is short-lived and can be controlled [59,60,78,88]. However, improved targeting moieties, with minimal off-target binding to reduce capillary leak syndrome, are still urgently needed.

A second problem of current bacterial toxin-based targeted delivery systems is the low efficiency of translocation during transmembrane cargo delivery. Currently, we know few details about the translocation of most bacterial toxins due to their dynamic nature, drastic structural rearrangements, as well as the involvement of the lipid bilayer environment. This highlights the need to study these mechanisms further.

Another problem of the current bacterial toxin-based delivery systems is the immune clearance of the drug. Because bacterial toxins are exogenous antigens, the immune systems of patients can recognize the toxin and neutralize it before it enters the targeting cells, which can significantly reduce the efficacy of these immunotoxins. Even though various mutants have been designed to reduce immunogenicity based on the study of the B cell and T cell epitopes as well as human neutralizing antibodies, advances in our understanding of immunology could help to design de-immunogenized versions of the bacterial toxins so that they have less clearance by anti-drug antibodies [89,90,91,92].

## 6. Future Directions

Capillary leak syndrome is one of the leading adverse effects of immunotoxin therapeutics. Although it can be partially controlled by proper medical management, it is still the dominant dose-limiting factor of ITs [78,88]. Capillary leak syndrome is initiated by the binding and damage to human endothelial cells by ITs. Previous studies have shown that toxin consensus structural motifs (x)D(y) are exposed to toxin surfaces and affect cell-cell interactions and damage endothelial cells, where x could be amino acid L, I, G, or V, and y could be amino acid V, L, or S. For example, the Diphtheria toxin A subunit contains two VDS motifs, while the PE38 toxin fragment has one GDV and two GDL motifs. The deletion or mutation of these (x)D(y) structural motifs without compromising IT efficacy is a successful approach in decreasing human endothelial cell damage and the resulting capillary leak syndrome [58,59,60]. Additionally, other motifs are involved in the nonspecific cell binding and the associated side effect of Its [78]. To gain a comprehensive understanding of the underlying etiology and origins of these side effects, it is crucial to identify targeted human cell lines and employ human models to mimic the capillary leak syndrome. Further studies involving cell surface toxin receptor screening, as well as toxin binding motif identification and modulation, could allow for the development of an improved version of ITs with reduced adverse effects stemming from non-specific binding [3,6].

Another major hurdle for IT-based drug delivery is the relatively low translocation efficiency across cellular membranes, as a significant portion of toxin molecules fail to reach the cytosol [93]. A mechanistic understanding of the bacterial toxin translocation process, especially the interplay between the toxin and lipid membrane during translocation, is crucial for realizing the potential of bacterial toxin-based immunotoxins. To unveil the structural and functional dynamics of bacterial toxins during translocation, high-resolution single-particle cryo-Electron Microscopy, single-molecule fluorescence resonance energy transfer (FRET), and electrophysiology, in combination with liposome and nanodisc lipid bilayer systems are needed to determine the high-resolution of structures and measure the functional dynamics of toxin translocation intermediates in detergents and a native-like lipid environment embedded in nanodiscs. Studying these structures could advance our comprehensive understanding of the spatial and temporal patterns of the protein cargo transmembrane delivery process of these toxins at the single-molecule level [10,94,95]. This could also contribute to the engineering and optimization of bacterial toxin translocation domains that can deliver cancer drugs into the sub-cellular compartment with enhanced efficacy of delivery [5]. Such an understanding could even establish a solid foundation to further design and engineer novel and programmable drug delivery systems for various intracellular protein-targeting drugs based on this naturally evolved and delicate protein delivery machinery [85].

The current ITs mainly utilize native toxic cargo to achieve cancer cell killing. Due to the highly modular nature of ITs, it is relatively easy to replace native toxic cargoes with other cargoes to finetune the intracellular pathways and cell-killing effects [4]. Phage-assisted evolution is another powerful approach to evolve toxin cargoes into enzymes with reprogrammed specificity. As a successful example, botulinum neurotoxin has been evolved by phage-assisted evolution to cleave the phosphatase and tensin homolog but not its native substrate in neurons [96]. It can be used to fine-tune the toxicity of the cargo and modulate vast intracellular cancer pathways to achieve precision cancer medicine.

Since bacterial toxin-based targeted delivery systems can specifically target cancer cells and kill them, such systems, with new antigenic targets, optimized translocation domains, fine-tuned toxic cargoes, and reduced off-target toxicity and immunogenicity, hold great promise to push the boundaries in developing novel treatments of cancers that remain incurable. The combination of immunotoxins with chimeric antigen receptor T cells (CAR-T), immune checkpoint blockade therapy, as well as anticancer nanoparticles can also create novel treatment opportunities for synergistic and superior anticancer outcomes [60,97,98,99,100].

## Figures and Tables

**Table 2:** Diphtheria toxin-based cancer prodrugs/drugs.

Toxin/Toxin Fragment	Targeting Moiety	Target Cancer Cells or Diseases	Obtained Outcome	References
DAB486	IL-2	CTCL, Hematological cancers, NHL	Significant tumor reductions in heavily treated patient group; half of the patients developed an antibody response to the toxin	[54,61]
DT389	IL-2	CTCL, Hematological cancers, NHL	Patients showed significant improvement in tumor response and quality of life	[56]
DT388	IL-3	BPDCN, AML	The drug exhibited potent cytotoxicity towards BPDCN and AML cell lines; the IT treatment showed robust clinical activity in patients with BPDCN	[17]
DAB389	EGF	Bladder cancer, lung cancer	Human bladder cancer lines showed specific and robust response with DAB389EGF treatment	[62]
DAB389	IL-7	Hematopoietic malignancies	DAB389IL-7 fusion protein is selectively cytotoxic for cells bearing the IL-7 receptor	[63]
DT389	IL-13	Glioblastoma	DT389IL-13 fusion protein resulted in significant tumor volume reduction and the significantly prolonged survival of mice with brain tumors	[64]
DT390	IL-13 and EGF bispecific ligand	Glioblastoma, prostate, and pancreatic cancer	DTEGF13 protein selectively killed human glioblastomas and showed a higher activity than its monospecific IT counterparts	[65]
DT390	CCR4 scFv	Glioblastoma, prostate, and pancreatic cancer	CCR4 IT depleted monkey CCR4(+) cells in vitro; around 80% CCR4(+)Foxp3(+) Tregs were depleted in the tested monkeys	[66]

**Table 3:** PE toxin-based cancer prodrugs/drugs.

Toxin/Toxin Fragment	Targeting Moiety	Target Cancer Cells or Diseases	Obtained Outcome	References
PE38	IL-4	Solid tumors, recurrent malignant glioma	Tumor necrosis following treatment in many patients	[82]
PE38	IL-13	Glioblastoma	The IT was well-tolerated but had no survival advantage compared with Gliadel wafers in a Phase III evaluation	[79]
PE38	Anti-CD22 antibody	Hairy cell leukemia	IT treatment resulted in rapid depletion of CD19(+)B cells and rapid reduction in tumor volume	[18]
PE24	Humanized anti-mesothelin Fab	Pancreatic adenocarcinoma	The drug showed antitumor activity in around half of the treated patients	[81]
PE38	Anti-EGFR antibody	Glioblastoma	ADA against the drug and capillary leak syndrome was seen as a dose-limiting factor	[83]
PE40	Lewis(Y) carbohydrate antigen targeting BR96 sFv	Lewis(Y)-positive metastatic carcinoma	The drug achieved prolonged survival in intracranial tumor models	[84]
PE252-608 fragment	Humanized anti-EpCAM single chain antibody	Non-muscle invasive bladder cancer	Complete response achieved in half of the patients with mild to moderate adverse effects that were treatable	[80]

## Data Availability

Not applicable.

## Abbreviations

ITs	immunotoxins
PA	protective antigen
LF	lethal factor
EF	edema factor
ANTXR1	anthrax toxin receptor 1
ANTXR2	anthrax toxin receptor 2
PA83	83-kDa PA monomer
PA63	63-kDa PA monomer
MAPKKs	mitogen-activated protein kinase kinases
cAMP	cyclic adenosine monophosphate
ADP	adenosine diphosphate
EGF	epidermal growth factor
EGFR	epidermal growth factor receptor
HER2	human epidermal growth factor receptor 2
HB-EGF	heparin-binding EGF-like growth factor
IL-2	interleukin-2
IL-2R	interleukin-2 receptor
IC50s	half maximal inhibitory concentrations
CD4+	CD4-positive
BPDCN	blastic plasmacytoid dendritic cell neoplasm
CR	complete response
PR	partial response
CTCL	cutaneous T-cell lymphoma
NHL	non-Hodgkin lymphoma
Treg	regulatory T cell
CCR4	CC chemokine receptor 4
AML	acute myeloid leukemia
PE	*Pseudomonas* exotoxin A
Foxp3	forkhead box p3
LRP1	low-density lipoprotein receptor-related protein 1
ER	endoplasmic reticulum
Fab	Fragment antigen-binding
EpCAM	Epithelial cell adhesion molecule
HCL	hairy cell leukemia
eCISs	extracellular contractile injection systems
EMA	European Medicines Agency
FDA	Food and Drug Administration
FRET	fluorescence resonance energy transfer
cryo-EM	cryo-Electron Microscopy
NAD	Nicotinamide adenine dinucleotide
EF-2	elongation factor-2
eEF-2	eukaryotic elongation factor-2
dsFv	disulfide stabilized variable fragment
CD22	cluster of differentiation22
CAR-T	chimeric antigen receptor T cells
